# Books and Magazines

**Published:** 1913-02

**Authors:** 


### Books and Magazines.

Practical Medicine Series. Vol. IX, 1912. Skin and Venereal Diseases. Edited by W. S. Baum, M. D., and H. N. Moyer, M. D., Chicago. The Year Book Publishers. Price, $1.25.

Volume X and last of the series of ten, issued at about monthly intervals each year, and covering the entire field of medicine and surgery. Each volume is complete for the year prior to its publication, on the subject of which it treats. The price of the set is $10, but any volume may be bought separately. Volume X is "Mental Diseases," Hugh Patrick, M. D., and Peter Bassoe, M. D., Editors. Price, $1.35.

---

The Chemic Problem in Nutrition (Magnesium Infiltration). A Sketch of the Causative Factors in Disorders of Nutrition as Related to Diseases of the Nervous System. By John Aulde, M. D. Cloth, 8vo, gilt top, pp. xvi, 410; 4 plate illustrations. Philadelphia, 1912. Price, $3, postpaid.

The task of the critic is both delicate and responsible. He must be sufficiently familiar with the subject matter to enable him to decide upon the correctness of the author's premises as well as the logic of his arguments and deductions. His responsibility arises as a result of his authoritative position, since he is supposed to protect his readers against sophistry and fallacious disputation.

In the present instance, notwithstanding the radical character of the teachings, the difficulties are more apparent than real, but this conclusion is reached only after a critical investigation of the claims advanced. For example, the volume has to deal almost exclusively with the effects of acids (acid excess) upon function and structure—and the gist of the argument aims to demonstrate that alkalescence of the body fluids (blood and lymph), being normal, acid excess necessarily leads to disorders of function, and ultimately to changes in structure, known as organic disease.

Figuratively, we might consider disease in the abstract as presenting an immense red (acid) background, through which irregularly appears a thin blue line of alkalescence, indicative of the enfeebled state of the vital activities. Then, by means of suitable chemicals, to neutralize or eliminate the acidity, the faint blue line expands until the color scheme is reversed—and we have faint streaks of red on a blue field. That is to say, under normal conditions, with a single exception, acid is an end product. An acid surplus, therefore, shows an accumulation in the system of waste material; hence, to restore function and maintain the integrity of structure, two methods are available—neutralization and elimination.

Here we find the first radical departure from modern methods, treatment by elimination having been found utterly useless—and harmful; useless because "elimination" of acid excess is neither practical nor possible, harmful because the so-called physiologic remedies employed are directed against *the effects,* and the thin blue line of alkalescence becomes less and less distinct and finally disappears.

It should be noted here that these teachings, in a measure, verify the adage, "There is nothing new under the sun." In the latter half of the seventeenth century was founded the iatro-chemical sect. According to Sylvius, an industrious student of Van Helmont and Descartes, health depends upon the relation of the fluids, acid and alkaline, their union producing a neutral and milder substance. Two kinds of diseases were distinguished, the result either of acid or alkaline acridity. Among the prominent followers of Sylvius might be mentioned Willis, the celebrated English anatomist; Glauber, the discoverer of sodium sulphate (Glauber's salt), and many others; but iatro-chemistry gradually lost repute, and was completely overthrown early in the eighteenth century, principally through the teachings of Hoffmann.

Founded upon mere assumption and a smattering of chemistry, with no definite conception of physiology, iatro-chemistry was foredoomed to failure. It seems paradoxical, too, because this was a period of exceptional activity in laying the foundations of medical science. Such names as Harvey, Steno, Vieussens, Malpighi, Spigelius, Bartholin, Asselius, Pauli, Mentel, Wesling, Highmore, Glisson, Wharton, Leeuwenhoeck, Ruysch, Sydenham, Boerhaave, Stahl, Albertini, Valsalva, Bellini, Swammerdam, Meibomus, Peyer, Duverney, Cowper, all belong to this period, and it is remarkable, to say the least, that they failed to distinguish the normal alkalinity of the building-up from the normal acidity incident to the breaking-down processes in both animal and plant life.

Running in parallel lines throughout this sketch, we find these biologic principles brought prominently to the fore—just as the keystone of the arch is to architecture, the flange on the wheel to commerce, so alkalescence (of the body fluids and tissues) is the pivot or turning point between health and disease.

The most conspicuous example of "radicalism," however, is to be found in the results attained in following up the working hypothesis. That is to say, acid excess—a convenient and expressive term used to designate a diminished alkalinity of the blood—being abnormal, we should endeavor to determine *the effects,* and this brings us to the all-important question, the chemic problem in nutrition.

The work is divided into three sections, mainly for the purpose of establishing a scientific basis for the rational treatment of diseases of the nervous system. The first two chapters—"General Considerations"—deal with the various questions relating to health maintenance, especially pointing out the insidious and progressive character of disorders arising from the chemic deviation—contrasting the unfortunate results of modern methods when compared with the efficiency and simplicity of treatment based upon the unerring laws of chemistry. The physical and chemic conditions leading to disease are clearly and intelligently discussed, so that the reader can easily determine the significance of the various sign-posts which point to immunity.

More in detail we find these questions answered in the section on "Disorders of Nutrition." Whether it be from the physiologic viewpoint, from a study of the peculiarly intricate processes incident to metabolism, or the enormous surface energy of inorganic ferments, the chemic deviation responsible for magnesium infiltration is never overlooked.

Naturally, the reader wants to know how magnesium has all at once become such a dangerous factor in modern civilization, and this question is satisfactorily solved in a separate chapter, entitled "The Food Problem, with Dietary Studies." It shows, for example, how a dietary containing magnesium in excess of the normal demands of the system gives rise to digestive derangements, defective secondary assimilation, with consecutive involvement of the nervous system, a parallel being found between pellagra and the injurious effects of magnesium on plant life.

A critical study of the chemic deviations in the vascular system —arising from factitious, inorganic deposits and appearing as "degenerations"—forms a suitable introduction to an important chapter, "The Causative Factor in Heart Failure," a malady which is rapidly increasing. The working hypothesis is completely developed in a chapter entitled "The Magnesia Heart," the description being both novel and ingenious; moreover, it is also scientific and practical. It is novel, because of its fundamental character, while the ingenuity lies in the application of well-known chemic principles relating to "mass action," "quartation" and "parting." Together with the case reports, it harmonizes clinical observation with scientific facts—it clears away, at a single stroke, the cobwebs of superstition along with the follies of tradition.

For example, we know that "heart action," as well as all organic function, depends upon integrity of the nerve supply; hence, acid excess leads to heart weakness. But it does even more than this. An excess of acid depletes the lime content of the tissues, including nerve tissue, when there is coincident or consecutive replacement by magnesia, a nonconductor of electricity—when the heart is insulated, or a "short circuit" arrests the transmission of nerve impulses. The treatment of heart weakness, therefore, is conducted for the sole purpose of promoting magnesium dissociation—to the end that normal innervation shall be re-established. Thus, there are three cardinal principles to be considered—(1) restore the digestive capacity; (2) neutralize acid excess; and (3) promote magnesium dissociation.

In addition to the details covering the treatment of typical cases, informative tabulations are interspersed through the text, so that the reader can readily find answers to any queries which may arise.

This review has already far exceeded the limits originally mapped out for it, while no reference has been made to magnesium infiltration, as the "Complication" in constitutional maladies, nor to "Diseases of the Nervous System," but the pathology and treatment differ in no essential respect. Thus, while magnesia heart, neuritis, neuralgia, Bright's disease, Jacksonian epilepsy, and infantile paralysis are localized or circumscribed manifestations of magnesium infiltration, neurasthenia, St. Vitus' dance (in childhood), "catarrh" and arterio-sclerosis may be classed as disseminated, but the cardinal principles of treatment remain unchanged.

A comprehensive index, analytic, synthetic and clinical, covering ten pages, makes consultation easy, and from a mechanical viewpoint the volume is a handsome specimen of the bookmaker's art.—*Contributed.*

